# Understanding Influenza Vaccination During Pregnancy in Canada: Attitudes, Norms, Intentions, and Vaccine Uptake

**DOI:** 10.1177/10901981211001863

**Published:** 2021-04-17

**Authors:** Devon Greyson, Ève Dubé, William A. Fisher, Jocelynn Cook, Manish Sadarangani, Julie A. Bettinger

**Affiliations:** 1 University of Massachusetts–Amherst, Amherst, MA, USA; 2 Institut national de santé publique du Québec, Quebec City, Quebec, Canada; 3 CHU de Québec-Université Laval, Quebec City, Quebec, Canada; 4 Western University, London, Ontario, Canada; 5 The Society of Obstetricians and Gynaecologists, Ottawa, Ontario, Canada; 6 University of British Columbia, Vancouver, British Columbia, Canada

**Keywords:** pregnancy, quantitative methods, theory of planned behavior, theory of reasoned action, vaccination and immunization

## Abstract

To improve uptake of influenza vaccine in pregnancy, it is important to understand the factors that predict prenatal vaccination. The aim of this study was to test the capability of the theory of planned behavior, augmented with information constructs, to predict and explain influenza vaccination uptake in a sample of 600 pregnant individuals in Canada. A baseline survey at the start of influenza season assessed beliefs, norms, perceived control, and information-seeking behavior related to influenza vaccination in pregnancy, as well as respondent demographics. A follow-up survey at the conclusion of influenza season assessed self-reported influenza vaccine uptake as well as infant vaccination intentions. Multivariable analysis indicated that attitudes toward influenza vaccination in pregnancy, subjective norms, information seeking, and past vaccination behavior predicted intentions to be vaccinated, and intentions predicted vaccine uptake. Neither perceived control nor demographics were significant predictors of intentions or vaccine uptake. These findings suggest that presumptive offering of vaccination in pregnancy by health care providers, as well as patient and public health educational interventions, may be effective in communicating norms and strengthening positive attitudes and intentions concerning influenza vaccination in pregnancy, resulting in higher vaccine coverage.

Pregnancy increases the risk of influenza-related morbidity and mortality (Dodds et al., 2007; Jamieson et al., 2009; Legge et al., 2014). Vaccination against influenza (flu) has been recommended during flu season for pregnant individuals in Canada since 2007 (National Advisory Committee on Immunization, 2015). Despite strong recommendations by North American medical and public health bodies, influenza vaccine uptake among pregnant women remains suboptimal and well below the recommended target of 80% (Halperin et al., 2014; Moniz & Beigi, 2014; Yudin et al., 2010). Before the 2009 H1N1 influenza pandemic, it was estimated that around 15% of pregnant women were vaccinated annually against seasonal influenza in Canada. Although an increase in influenza vaccination uptake was observed during the H1N1 pandemic and the 2010–2011 flu season, this increase in prenatal influenza vaccine uptake has not been sustained in subsequent seasons (Halperin et al., 2014).

Because pregnant women are at a higher risk for influenza, and because their newborn infants are also at higher risk for influenza and other vaccine preventable diseases such as pertussis, it is possible to protect them through vaccination during pregnancy—a growing strategy for improved maternal–child health outcomes (Madhi et al., 2014; Moniz & Beigi, 2014). In addition to influenza vaccine, the pertussis vaccine has recently been recommended in Canada during every pregnancy (Brophy et al., 2018; Castillo & Poliquin, 2018), and new vaccines that may be licensed for prenatal administration (e.g., to protect against Group B streptococcus and respiratory syncytial virus, which are presently in clinical trials) may be available in the future. For vaccination in pregnancy to be a viable strategy, however, we must understand more about pregnant individuals’ vaccination beliefs and actions.

Identifying determinants of influenza vaccine uptake in pregnancy may help target education efforts and intervention strategies to most efficiently promote vaccination in pregnancy. Previous studies have identified patient-level factors associated with acceptance of influenza vaccination during pregnancy in Canada, including perception of disease susceptibility and severity, perception of benefits—including safety and effectiveness—of vaccination, being cared for in pregnancy by a family practitioner, greater knowledge about influenza and vaccinations, and demographic factors including multiparity, comorbidities, higher income, higher education, married status, and older maternal age (Poliquin et al., 2019). Some studies have applied the health belief model (Bettinger et al., 2016; Fabry et al., 2011), but most have not been theory driven, instead using cross-sectional data to identify demographic factors associated with vaccination (Brien et al., 2012; Gracie et al., 2011; Hilderman et al., 2011; Legge et al., 2014; Liu et al., 2012; Yudin et al., 2009), or exploring of the knowledge, attitudes, and beliefs of mothers (Halperin et al., 2014; Kowal et al., 2015), practitioners (Desjardins et al., 2017; Lee et al., 2005), or both (Tong et al., 2008). Although the theory of reasoned action (TRA) and related theory of planned behavior (TPB) have been useful in understanding vaccine intentions and behaviors for other vaccines, including human papillomavirus (HPV) vaccination in Canada and the United States (Catalano et al., 2017; Fisher et al., 2013; Roberto et al., 2011), and have been used in several U.S. studies of predictors of influenza vaccination in pregnancy, they have not to our knowledge been used to guide an investigation of predictors of influenza vaccination in pregnancy in Canada, where health system and sociocultural factors differ from the United States in important ways. Additionally, none of the existing TRA/TPB-based studies of vaccination in pregnancy have tested the addition of information behaviors (e.g., seeking or encountering information) as an augmenting construct, which has been found to improve predictive power of the theories when applied to the topic of HIV testing (Meadowbrooke et al., 2014).

The current study examined psychosocial determinants of pregnant Canadians’ intentions and behavioral outcomes related to influenza vaccination during pregnancy from the perspective of the TPB. We also assessed the utility of augmenting the TPB with information behavior constructs in the prediction of vaccination intentions and behavior and assessed the relationship of intentions to behavior (influenza vaccine during pregnancy). The aim of this study was to identify potentially modifiable factors that influence pregnant Canadians to obtain seasonal influenza vaccination and to inform clinical and public health interventions to improve acceptance of recommended pregnancy vaccines. Since vaccination behavior during pregnancy has also been associated with infant and child vaccination (Robison & Osborn, 2017), this may have implications for vaccine confidence into and beyond infancy.

## Method

This study involved two sequential online surveys administered to individuals who were members of a preexisting survey panel. The first survey, taken during pregnancy (any trimester) and at the start of flu season in Canada (October), asked about the intention to receive influenza vaccination during pregnancy and contained questions about potential determinants of vaccination (attitudes, social norms, perceived behavioral control, and health information behavior) as well as participant demographics that may act as predictors (e.g., parity, age, education), to assess associations among these potential determinants and influenza vaccination intentions. The second survey followed up with the same participants at the end of flu season, asking about influenza vaccination behaviors since the first survey, as well as brief direct measures of attitudes, social norms, and perceived behavioral control regarding vaccination of their infant, to assess associations among the first survey’s potential determinants and intentions, and self-reported vaccination in pregnancy, and between these and infant vaccination intentions and self-reported behavior. All research procedures and documents were approved by the Research Ethics Board of the University of British Columbia.

### Theoretical Approach

This study’s theoretical grounding was the TPB (Ajzen, 1991), which has been widely used to predict health-related behaviors. TPB’s efficiency and validity is recognized for general health behaviors (Montaño & Kasprzyk, 2008) as well as specifically for vaccination-related behaviors (Godin & Kok, 1996). The TPB is an extension of the TRA; both theories posit that intentions are a strong predictor of health behaviors and that *intentions* are determined by individuals’ *attitudes* (based on beliefs about the behavior and likely outcomes resulting from it), and *perceived social norms* (based on perceptions of whether others think one should engage in the behavior and how much those opinions matter to the individual). The TPB additionally suggests *perceptions about the individual’s behavioral control*, or ability to carry out the behavior in question, is a third determinant of action. Surveys informed by the TRA or TPB typically assess these constructs with a series questions requesting a response using 7-point adjective scales (e.g., good–bad or easy–difficult; see Supplemental Appendix A, for full survey questions and scales used in the current survey). Effective intervention efforts can be informed by knowing whether intentions or behavior are under attitudinal, normative, behavioral control, or joint control, and by understanding the specific modifiable belief underpinnings of relevant attitudes, norms, and control beliefs. This survey used both direct measure questions and indirect questions about respondents’ beliefs, which were developed based on previous elicitation research by research team members.

**Figure 1.**
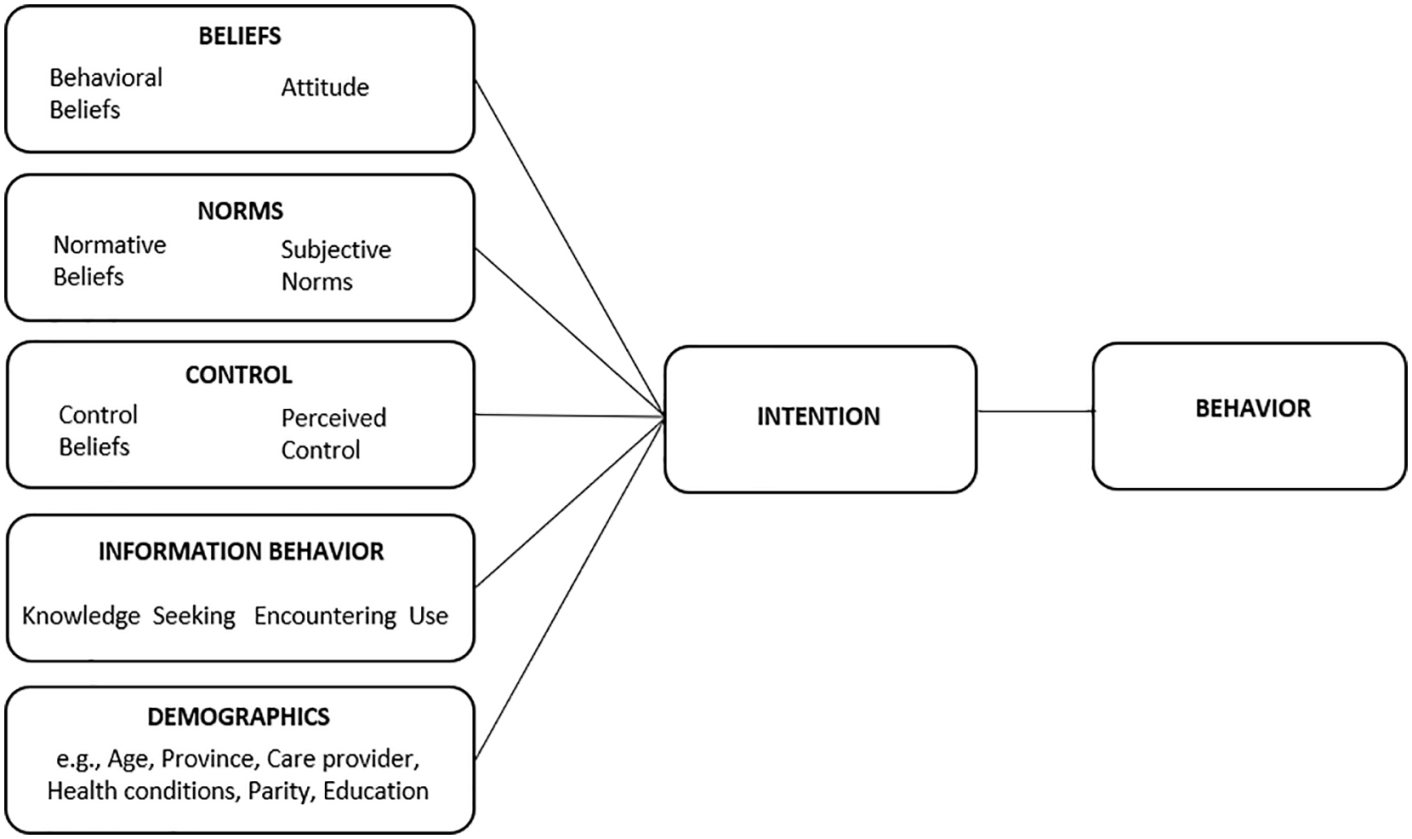
Theoretical model of predictors of prenatal influenza vaccination and behavior.

A number of modifications to the TPB have added information-related constructs; for example, the integrated behavioral model (Montaño & Kasprzyk, 2008) proposes that informational constructs such as past experience and level of knowledge have indirect effects on intention, and the information-augmented TPB (Meadowbrooke et al., 2014) found that including certain measures of *information behavior* (e.g., information seeking, encountering, and use) improves the predictive power of the TPB. In the current study, TPB was augmented with information seeking and utilization behavior constructs (TPB+i) in an attempt to improve predictive validity of the model and identify the most influential determinants of vaccination intention and behavior. Figure 1 depicts the hypothesized model of predictors of influenza vaccination in pregnancy.

### Data Collection

This study prospectively collected data by bilingual (French and English, the official languages of Canada) online survey in a two-phase process, with invitations sent to a panel population (administered by Logit group, Toronto, Ontario, Canada) that was geographically representative of the Canadian population based on the 2011 Canadian census profile (Government of Canada, 2012). Consent was obtained using double opt-in recruitment procedures (to join the research panel and to respond to the particular survey seeking pregnant panel members). Incentives were offered by the panel company in accordance with their practices (e.g., offering “points” toward retailer rewards), with substantially increased incentives offered in an effort to retain participants at follow-up. Phase 1 surveyed 600 pregnant individuals in October 2017 to assess the strength of association among information-augmented TPB (TPB+i) constructs and pregnant individuals’ intentions to receive the influenza vaccine. The number of participants (600 for Phase 1 survey) was based on an anticipated recall rate of 35% for follow-up, to provide a sample size of 210 for Phase 2, roughly in line with the sample sizes of other TRA/TPB-based vaccination studies with significant findings (Fisher et al., 2013; Myers, 2016). Respondents were assigned a unique participant ID number, which was used to resample the same respondent population for follow-up and to link responses from the same individual for analysis. Phase 2 took place after the Canadian influenza season in late April to early May 2018, by recontacting Phase 1 respondents and asking them to report whether they had been vaccinated against influenza, as well as their intentions for vaccinating their infants. Self-report of current-season influenza vaccine receipt is generally accurate (≥90% agreement with vaccine registry data), with high sensitivity and specificity, although recall of past seasons’ vaccination may wane with time (Irving et al., 2009; King et al., 2018).

A longitudinal study design is recommended when using TPB, with the predictor constructs (attitudes, norms, perceived control, and information seeking and utilization behaviors) and intentions measured at one time point, and the behavioral outcomes measured after a reasonable time interval. The survey instruments were designed using findings elicited in the researchers’ previous studies of vaccine hesitancy among pregnant and parenting Canadian women (Bettinger et al., 2016; Dubé et al., 2016, 2019). Surveys were pilot tested in both languages, and soft launched to test for technical issues and face validity, prior to full survey launch. The Phase 1 survey consisted of 67 questions. The first 50 used a 7-point scale to measure TPB+i constructs; additional multiple-choice questions collected relevant participant demographics in a standard format used by the Society of Obstetricians and Gynaecologists of Canada (to make possible comparisons with other Society of Obstetricians and Gynaecologists studies). The Phase 2 survey consisted of seven questions plus reiteration of demographic questions from Survey 1. Survey instruments are available in Supplemental Appendix A.

### Analysis

We used individual and composite variables to create descriptive statistics to characterize participants, their vaccination intentions, and their reported behavior. Data analysis took place using SAS Version 9.4 for Windows. Internal consistency of multiple-item constructs was calculated using the Cronbach alpha coefficient, and items were retained when the coefficient was greater than .65. Correlations between an item and the corrected overall rating of the construct (without the score of the item) were also calculated.

Univariate analyses were conducted for Survey 1 and Survey 2 separately. First, correlation analysis was conducted to assess the relationships between participant demographics, attitudes toward influenza vaccination in pregnancy, perceived norms regarding influenza vaccination in pregnancy, perceived ability to obtain influenza vaccination in pregnancy, and the Survey 1 outcome: a single question assessing intention to get the influenza vaccine (commonly referred to as “flu shot”) during the current pregnancy (“I will get a flu shot this season while I am pregnant,” rated on a 1–7 scale of likelihood). Then, correlation analysis was conducted between the Survey 1 predictors and intention outcome, and the categorical outcome for Survey 2, self-reported behavior (“Did you receive a flu shot this past fall/winter (2017/2018)?” answered with Yes, No, or Unsure). While we analyzed how the samples for Survey 1 and Survey 2 were different, we did not use any imputation to try to address loss to follow-up.

### Multivariable Regression Models

Multiple regression analysis was used to determine the unique contribution of the constructs and variables of interest as predictors of intention and behavior. In the analysis of Survey 1, respondent intention to get vaccinated for seasonal influenza during the current pregnancy (on a scale of 1–7) was treated as the dependent variable. Independent variables included beliefs (direct measures of attitude and indirect measures of behavioral beliefs), subjective norms (direct measure of norms and perceived norms), perceived behavioral control (direct measure of perceived control and control beliefs), informational constructs (knowledge, information seeking, encountering, and use for vaccine decisions), and demographic attributes (including sociodemographic, obstetric and health history including parity, and reported previous influenza vaccination behavior). Regression models (linear for intention, rated on a scale; logistic for behavior, with dichotomous response) were built using a stepwise procedure. To be included, a variable, including sociodemographic variables, was required to be significant at a level of *p* ≤ .20. Only those variables that remained significant at a *p* ≤ .05 level were retained in the final model, including sociodemographic variables. This procedure was repeated for Survey 2, but the behavior (influenza vaccination, dichotomized into Yes vs. No/Unsure) was treated as the dependent variable.

## Results

### Study Population

The initial survey population comprised 600 pregnant Canadians and 84 (14%) of Survey 1 respondents completed the follow-up Survey 2. The Survey 2 respondent population (a subset of Survey 1) was on average a little older than that for Survey 1, including a greater percentage of 35- to 44-year-olds and relatively fewer in the 18- to 24-year-old bracket. Otherwise, the survey populations were similar in measured demographics, including geography, ethnicity, perinatal health care provider types, parity, chronic health conditions, income, and education. Table 1 presents a demographic summary of the study populations. The study population had a greater number of respondents under 25 years of age than would be expected, as 13.4% of Canadian live births in 2017 were to mothers aged 15 to 24 years; otherwise the ages were representative of maternal ages in the Canadian population (Government of Canada, 2018). Compared with the general Canadian population reported in the 2016 census (Government of Canada, 2017), the geographic distribution of respondents was representative of the national population, as was the percentage of the study population born outside Canada, and the reported educational attainment of the study population was close to that of the female Canadian population; however, the study population was more affluent, with only 45.5% of survey respondents reporting household income less than $60,000, compared with 81.5% of female census respondents.

**Table 1.** Study Population Characteristics for Survey 1 and Survey 2.

Demographic	Survey 1		Survey 2	
	*n*	%	*n*	%
Age (years)				
18–24	136	22.67	13	15.48
25–29	169	28.17	22	26.19
30–34	150	25.00	21	25.00
35–39	118	19.67	23	27.38
40–44	27	4.50	5	5.95
Province/territory				
British Columbia	80	13.33	14	16.67
Alberta	59	9.83	7	8.33
Saskatchewan	16	2.67	2	2.38
Manitoba	21	3.50	1	1.19
Ontario	231	38.50	43	51.19
Quebec	146	24.33	13	15.48
New Brunswick	14	2.33	1	1.19
Nova Scotia	18	3.00	1	1.19
Newfoundland	10	1.67	2	2.38
Northwest Territories	2	0.33	0	0
Prefer not to answer	3	0.50	0	0
Ethnicity				
European	253	42.17	38	45.24
First Nations, Metis, or Inuit	24	4.00	2	2.38
Chinese	30	5.00	6	7.14
South or Southeast Asian	37	6.17	9	10.71
Multiple	51	8.50	4	4.76
Other (aggregated)	150	25.00	22	26.19
Prefer not to answer	55	9.17	3	3.57
Pregnancy trimester (at Survey 1 time point)				
First (1–12 weeks)	238	39.67	29	34.52
Second (13–27 weeks)	235	39.17	37	44.05
Third (28+ weeks)	127	21.17	18	21.43
Pregnancy care provider (at Survey 1 time point; not mutually exclusive)				
Family physician	389	64.83	54	64.29
Obstetrician/gynecologist	341	56.83	47	55.95
Midwife	70	11.67	7	8.33
Other/prefer not to answer	24	0.04	3	3.57
Previous births (at Survey 1 time point)				
0	279	46.50	39	46.43
1	195	32.50	27	32.14
2	71	11.83	10	11.90
3	30	5.00	4	4.76
4	19	3.17	3	3.57
Prefer not to answer	6	1.00	1	1.19
Chronic health condition				
Yes	115	19.17	16	19.05
No	485	80.83	68	80.95
Formal education				
Secondary diploma or below	102	17.00	11	13.10
Some or completed college/university	346	57.67	55	65.48
Postgraduate studies	151	27.17	18	21.43
Prefer not to answer	1	0.17	0	0
Living situation				
Live with partner	454	75.67	61	72.62
All others	146	24.33	23	27.38
Where born				
Canada	499	83.17	68	80.95
Outside of Canada	93	15.50	16	19.05
Prefer not to answer	8	1.33	0	0
Employment				
Part time	22.67	22.67	19	22.62
Full time	54.33	54.33	51	60.71
No paid work	21.17	21.17	13	15.48
Prefer not to answer	1.83	1.83	1	1.19
Household income ($)				
<60,000	273	45.50	37	44.05
60,000+	299	49.83	44	52.38
Prefer not to answer	28	4.67	3	3.57

### Model Constructs and Univariate Correlations

Correlation analysis resulted in combined attitudes and combined behavioral belief constructs, single-question measures of direct and descriptive norms, as well as control beliefs, and combined constructs of information seeking, information use, and knowledge. To provide transparency into how our model was constructed, Supplemental Appendix B contains the model constructs, including questions that showed sufficiently high correlation, as well as results of the univariate analysis of Survey 1 (in which outcome = intention to be vaccinated in a linear regression). Within beliefs, the three attitude questions were used to build one *combined attitudes* construct. One pair of indirect behavioral belief questions (Questions 6 and 12; see Supplemental Appendix A for all questions) was discarded due to low correlation with the rest of the construct, leaving a *combined behavioral beliefs* construct comprising five question pairs. Within norms, the pairs of indirect normative belief questions (18–21 and 23–26) did not correlate well and the construct was therefore discarded, leaving the two direct measures of *direct norms* and *descriptive norms* for use in our analysis. The questions assessing control beliefs and perceived behavioral control did not show sufficient correlation to build compound construct (Questions 30–35). Therefore, a single, simple direct measure alone was retained to represent *control* in the model. Within information, combined *information seeking* was composed of three questions, but information encountering construct was dropped due to low correlation of the questions. A combined *information use* construct was built of four questions, and *knowledge* was constructed with four correlated questions, with a fifth noncorrelated question (Question 48) discarded.

In our univariate correlation analysis of Survey 1, we found stronger intention to receive the influenza vaccine in pregnancy to be associated with attitudes, behavioral beliefs, direct and descriptive norms, control, information seeking, information use, knowledge, past receipt of influenza vaccine, having completed postsecondary degree, and working full time versus not working for pay.

Fewer constructs were determined to be significantly associated with Survey 2’s outcome of having received the vaccine (behavior) at a level making them eligible for inclusion in the multivariable analysis. Those that were included intention (the dependent variable in Survey 1), attitude, behavioral beliefs, direct and descriptive norms, and previous year receipt of influenza vaccine. Table 2 shows these results.

**Table 2.** Univariate Correlation Analysis of Survey 2 (Behavior Outcome).

Variable	Univariate correlation estimate	95% CI	*p*
Intention to vaccinate	**0.60**	**[0.46, 0.78]**	**<.001**
Combined attitudes	**0.61**	**[0.45, 0.81]**	**<.001**
Combined behavioral beliefs	**0.93**	**[0.88, 0.99]**	**.032**
Direct norms	**0.65**	**[0.51, 0.84]**	**<.001**
Descriptive norms	**0.67**	**[0.49, 0.91]**	**.011**
Control	1.07	[0.84, 1.35]	.590
Information seeking	0.77	[0.57, 1.04]	.089
Information use	1.00	[0.76, 1.31]	.980
Knowledge	0.79	[0.56, 1.11]	.177
Last year behavior (Yes vs. No)	**5.93**	**[2.09, 16.85]**	**<.001**
Weeks pregnant	1.02	[0.98, 1.07]	.323
Previous births (1 vs. none)	0.82	[0.30, 2.23]	.934
Previous births (2 vs. none)	0.84	[0.20, 3.49]	.993
Previous births (3 vs. none)	0.56	[0.07, 4.42]	.630
Previous births (4+ vs. none)	1.12	[0.09, 13.48]	.780
Age (25–29 years vs. 18–24 years)	0.95	[0.22, 4.19]	.778
Age (30–34 years vs. 18–24 years)	0.59	[0.14, 2.55]	.446
Age (35–39 years vs. 18–24 years)	0.41	[0.10, 1.71]	.101
Age (40–44 years vs. 18–24 years)	1.78	[0.15, 21.39]	.410
Education (college/university vs. secondary or less)	1.16	[0.32, 4.26]	.623
Education (postgraduate vs. secondary or less)	2.17	[0.45, 10.44]	.261
Where born (Canada vs. other)	0.29	[0.08, 1.12]	.073
Working (part time vs. not working)	2.80	[0.65, 12.09]	.158
Working (full time vs. not working)	1.43	[0.44, 4.68]	.736

*Note*. Values in boldface indicate statistical significance.

### Multivariable Regression Analysis

In the multivariable model, directly expressed positive attitude toward influenza vaccination in pregnancy was the strongest predictor (multivariable regression estimate 0.82; 95% confidence interval [CI] [0.75, 0.89]; *p* < .001) of intention to receive influenza vaccination in the current pregnancy, followed by having always received the influenza vaccine in the past 5 years (−0.47; 95% CI [−0.86, 0.10]; *p* < .001). Having sought information (0.12; 95% CI [0.06, 0.19]; *p* < .001), and direct norms (0.10; 95% CI [0.04, 0.16]; *p* < .004) were weaker predictors of intention to be vaccinated. Table 3 shows the results of the multivariable regression analysis of Survey 1.

**Table 3.** Results of Multivariable Regression Analysis on Intention Outcome.

Variable	Question No.(s)	*R*^2^ change	Multivariable regression estimate	95% CI	*p*
Combined attitudes	2, 3, 4	**.7110** ^a^	**0.82**	**[0.75, 0.89]**	**<.001**
Direct norms	17	**.0045**	**0.10**	**[0.04, 0.16]**	**<.004**
Control	29	.0012	0.04	[−0.02, 0.09]	.143
Information seeking	37, 38, 45	**.0186**	**0.12**	**[0.06, 0.19]**	**<.001**
Last year behavior	52 (Yes vs. No)	.0010	−0.26^b^	[−0.55, 0.02]	.180
	52 (Not sure vs. No)	.0011	−0.21^b^	[−0.68, −0.26]	.152
Past 5 years behavior	53 (Every year vs. Never)	**.0083**	−**0.47**^b^	**[**−**0.86,** −**0.10]**	**<.001**
	53 (Most–some–few years vs. Never)	.0075	−0.52^b^	[−0.78, −0.25]	<.001
Previous births	56 (1 vs. none)	.0013	0.12	[−0.08, 0.32]	.131

*Note.* Values in boldface indicate statistical significance.

a Model *R*^2^ prior to inclusion of other variables. ^b^Due to question wording, the result appears negative (see Supplemental Appendix for question wording). In all cases, past nonreceipt was associated with lesser intention to receive in current year.

**Figure 2.**
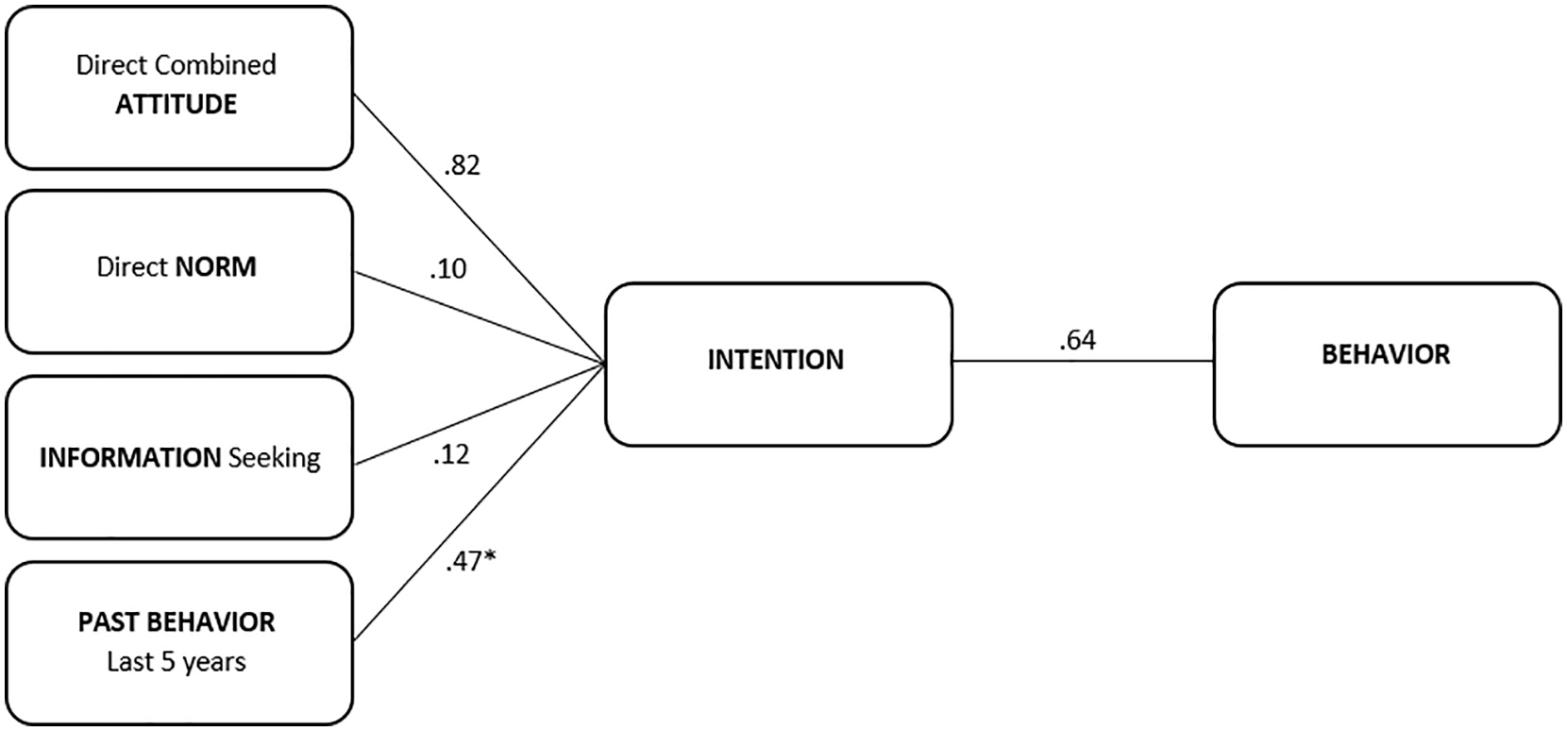
Final model of predictors of prenatal influenza vaccination intention and behavior. ^*^Due to the phrasing of the questions for survey usability, the output of this analysis was −.47; however, the relationship construct is presented here as positive to indicate strength of construct association without confusing directionality.

In the multivariable analysis of Survey 2, intention to be vaccinated was the only significant predictor for influenza vaccination in pregnancy (0.64; 95% CI [0.48, 0.85]; *p* = .002). Figure 2 depicts the revised model showing only the significant predictors remaining after multivariable regression analysis.

## Discussion

This study surveyed 600 pregnant Canadians at the beginning of the 2017 Canadian influenza season, to assess their self-reported beliefs, social norms, perceived behavioral control, information behaviors, and intentions related to influenza vaccination in pregnancy. Using the TBP, in the context of multivariable analysis, we found attitudes and norms, but not perceived behavioral control, to be correlated with intention to receive influenza vaccine that year. This suggests that the TRA (which does not contain the perceived control construct, but is otherwise similar to TPB) may be a better fit for predicting influenza vaccine intentions in pregnancy, echoing previous findings regarding young adult HPV vaccination (Fisher et al., 2013). Multivariable analysis also assessed the predictive utility of augmenting the TPB/TRA with information constructs. Among the four information constructs we tested (information seeking, encountering, use, knowledge), information seeking was the only one that was significantly associated with intention to receive influenza vaccine. Although some research has indicated that active vaccine information seeking is associated with vaccine hesitancy, this relationship is not necessarily the case for influenza vaccine in pregnancy. Among the demographics we collected from participants, none were predictive of intention to receive the vaccine for the coming influenza season, despite previous studies with other samples that found attributes such as age, income, chronic health issues, and type of perinatal health care practitioner to predict influenza vaccination in pregnancy. However, report of past receipt of the influenza vaccine was predictive of intention and receipt in the current season. In the follow-up survey, as was anticipated based on the TPB/TRA, attitudes toward vaccination during pregnancy and past vaccination behavior were correlated with intentions to receive the vaccine as assessed in October, which were in turn correlated with reported receipt of the vaccine by the following April.

### Limitations

A key limitation of the current study was the low participation (*n* = 84, 14%) in the follow-up survey. While this did not affect our analysis of the core TPB constructs, it did limit the conclusions we were able to draw, as we were left with insufficient statistical power to assess additional questions such as association between prenatal vaccine intentions or receipt and subsequent infant vaccination intentions or receipt. Due to the substantial rate of loss to follow-up, and lack of ability to know what unobserved variables might differ between those who did and did not respond to the Survey 2 invitation, we did not use imputation to try to conduct these planned analyses. Furthermore, the sample being an online panel, while ensuring certain measures of representativeness for the general population (e.g., province, age, income, trimester of pregnancy), likely introduces biases along other axes (e.g., digital and information access), limiting generalizability of these findings.

### Implications

The current study carries implications for both research and practice. The results of this study reinforce previous findings (Bettinger et al., 2016; Fisher et al., 2013; Gorman et al., 2012) that attitudes and social norms, and the beliefs on which these are based, are key influences on adult vaccination intentions and behavior. Regarding clinical practice, these findings not only suggest that screening on the basis of demographics is unlikely to be fruitful in predicting influenza vaccine acceptance in pregnancy but also indicate complex screenings are unnecessary. Rather, asking whether a patient usually obtains seasonal influenza vaccination, and what their intentions are toward influenza vaccination in the current year, possibly on an intake form, would help identify those likely to accept vaccination without additional persuasion and those potentially requiring time and attention to discuss risks and benefits in their specific case. To reinforce vaccination in pregnancy as a social norm, we recommend that perinatal health care providers adopt a presumptive, or announcement, approach to vaccine counseling that has been found to be successful in pediatric vaccination settings (Brewer et al., 2017; Opel et al., 2013), particularly with patients who have previously received influenza vaccination and indicate positive intentions toward doing so during pregnancy. This approach involves announcing that it is time for a vaccine (e.g., “Flu season is starting, so it’s time to get the influenza vaccine”) and only addressing specific concerns or offering additional time to consider the decision if concerns are raised by patients. If patients have been screened and are known not to have received influenza vaccination in the recent past, and not to intend to receive it during pregnancy, they may require more of a conversation to address their hesitancy, which would likely focus on providing information on safety of influenza vaccine during pregnancy as well as benefits to pregnant person, fetus, and eventual infant, in order to shift attitudes toward vaccination in a positive direction. Importantly, clinicians should avoid shaming patients for seeking out vaccine information on their own, as information seeking here was found to be associated with receipt of vaccination, despite sometimes being thought to increase antivaccine misinformation. Public health campaigns may also play a role in setting the norm of influenza vaccination in pregnancy, for example, with annual flu shot advertisements. To improve attitudes toward vaccination in pregnancy, prenatal classes and information resources can address common influenza vaccination questions and concerns and share evidence on the safety of the vaccine for pregnant individuals and their fetuses.

Regarding theory, the validation of some but not all of the information constructs proposed by Meadowbrooke et al. (2014) suggests that information remains a construct worth considering and may improve predictive validity of the TRA or TBP, but that more research is required to assess which constructs and examine whether information behaviors may be more predictive for some health behaviors (e.g., those that are logistically simple, but may entail a difficult decision-making process, such as vaccination or HIV testing) than others (e.g., those that are less deliberative but difficult to sustain over time, such as exercise or dietary change).

## Conclusion

Attitudes toward the influenza vaccine in pregnancy, and reported past receipt of influenza vaccine in recent years, appear to be the strongest predictors of influenza vaccination intention and receipt in pregnancy, with social norms and information seeking on the topic also significantly associated. The TRA, potentially augmented with an information seeking construct, was found to fit as a model of influenza vaccination in pregnancy.

## Supplemental Material

sj-docx-1-heb-10.1177_10901981211001863 – Supplemental material for Understanding Influenza Vaccination During Pregnancy in Canada: Attitudes, Norms, Intentions, and Vaccine Uptake Supplemental material, sj-docx-1-heb-10.1177_10901981211001863 for Understanding Influenza Vaccination During Pregnancy in Canada: Attitudes, Norms, Intentions, and Vaccine Uptake by Devon Greyson, Ève Dubé, William A. Fisher, Jocelynn Cook, Manish Sadarangani and Julie A. Bettinger in Health Education & Behavior

sj-docx-2-heb-10.1177_10901981211001863 – Supplemental material for Understanding Influenza Vaccination During Pregnancy in Canada: Attitudes, Norms, Intentions, and Vaccine Uptake Supplemental material, sj-docx-2-heb-10.1177_10901981211001863 for Understanding Influenza Vaccination During Pregnancy in Canada: Attitudes, Norms, Intentions, and Vaccine Uptake by Devon Greyson, Ève Dubé, William A. Fisher, Jocelynn Cook, Manish Sadarangani and Julie A. Bettinger in Health Education & Behavior
