# Supplementary material for: Understanding Influenza Vaccination During Pregnancy in Canada: Attitudes, Norms, Intentions, and Vaccine Uptake
Source: Health Educ Behav. 2021 Apr 17;48(5):680–9. doi: 10.1177/10901981211001863 (PMC12675836; doi:10.1177/10901981211001863)
Supplement: sj-docx-1-heb-10.1177_10901981211001863 – Supplemental material for Understanding Influenza Vaccination During Pregnancy in Canada: Attitudes, Norms, Intentions, and Vaccine Uptake [file sj-docx-1-heb-10.1177_10901981211001863.docx]

**Appendix: Survey Questions**

**Survey 1**

**Please take a few minutes to answer these questions about your thoughts and plans regarding flu shots in pregnancy.**

**Choose the number that corresponds with your answer.**

**For example, if the question was “How good do you think chocolate tastes?” and you think Chocolate is Very Good, you might select #1 like this:**

| Very good | 1 | 2 | 3 | 4 | 5 | 6 | 7 | Very bad |
| --- | --- | --- | --- | --- | --- | --- | --- | --- |

**Some of the questions might seem awkward or like they do not apply to you. Do your best to answer the questions according to how you feel or what you plan to do.**

**What are your thoughts and plans about getting a flu shot this season, while you are pregnant?**

| 1. I will get a flu shot this season while I am pregnant | | | | | | | | |  |
| --- | --- | --- | --- | --- | --- | --- | --- | --- | --- |
| Very likely | 1 | 2 | 3 | 4 | 5 | 6 | 7 | Very unlikely | Prefer not to answer |

| 2. Getting a flu shot while I am pregnant would be | | | | | | | | |  |
| --- | --- | --- | --- | --- | --- | --- | --- | --- | --- |
| Very good | 1 | 2 | 3 | 4 | 5 | 6 | 7 | Very bad | Prefer not to answer |
| 3. Getting a flu shot while I am pregnant would be | | | | | | | | |  |
| Wise | 1 | 2 | 3 | 4 | 5 | 6 | 7 | Foolish | Prefer not to answer |
| 4. Getting a flu shot while I am pregnant is | | | | | | | | |  |
| Necessary | 1 | 2 | 3 | 4 | 5 | 6 | 7 | Unnecessary | Prefer not to answer |
| 5. Getting a flu shot while I am pregnant would help me avoid the flu | | | | | | | | |  |
| Very likely | 1 | 2 | 3 | 4 | 5 | 6 | 7 | Very unlikely | Prefer not to answer |
| 6. Getting a flu shot while I am pregnant would help my baby to avoid the flu when it is a newborn | | | | | | | | |  |
| Very likely | 1 | 2 | 3 | 4 | 5 | 6 | 7 | Very unlikely | Prefer not to answer |
| 7. Getting a flu shot while I am pregnant would help protect other people in my community | | | | | | | | |  |
| Very likely | 1 | 2 | 3 | 4 | 5 | 6 | 7 | Very unlikely | Prefer not to answer |
| 8. Getting a flu shot while I am pregnant would make me sick | | | | | | | | |  |
| Very likely | 1 | 2 | 3 | 4 | 5 | 6 | 7 | Very unlikely | Prefer not to answer |
| 9. Getting a flu shot while I am pregnant could be bad for my baby | | | | | | | | |  |
| Very likely | 1 | 2 | 3 | 4 | 5 | 6 | 7 | Very unlikely | Prefer not to answer |
| 10. How likely is it that I will get the flu while pregnant? | | | | | | | | |  |
| Very likely | 1 | 2 | 3 | 4 | 5 | 6 | 7 | Very unlikely | Prefer not to answer |
| 11. Avoiding the flu while pregnant would be | | | | | | | | |  |
| Very good | 1 | 2 | 3 | 4 | 5 | 6 | 7 | Very bad | Prefer not to answer |
| 12. My newborn getting the flu would be | | | | | | | | |  |
| Very good | 1 | 2 | 3 | 4 | 5 | 6 | 7 | Very bad | Prefer not to answer |
| 13. Protecting other people in my community from the flu would be | | | | | | | | |  |
| Very good | 1 | 2 | 3 | 4 | 5 | 6 | 7 | Very bad | Prefer not to answer |
| 14. Having a side effect from the flu shot would be | | | | | | | | |  |
| Very good | 1 | 2 | 3 | 4 | 5 | 6 | 7 | Very bad | Prefer not to answer |
| 15. My baby being harmed by something I did in pregnancy would be | | | | | | | | |  |
| Very good | 1 | 2 | 3 | 4 | 5 | 6 | 7 | Very bad | Prefer not to answer |
| 16. Getting the flu while pregnant would be | | | | | | | | |  |
| Very good | 1 | 2 | 3 | 4 | 5 | 6 | 7 | Very bad | Prefer not to answer |

**What do people in your life think about getting a flu shot while you are pregnant?**

| 17. Most people who are important to me think I should get vaccinated against the flu during my pregnancy | | | | | | | | | |  | |
| --- | --- | --- | --- | --- | --- | --- | --- | --- | --- | --- | --- |
| True | | 1 | 2 | 3 | 4 | 5 | 6 | 7 | False | Prefer not to answer | |
|  | | | | | | | | | |  | |
| 18. My doctor or midwife thinks that I should get a flu shot in pregnancy | | | | | | | | | |  | |
| True | | 1 | 2 | 3 | 4 | 5 | 6 | 7 | False | Prefer not to answer | |
| 19. My partner thinks that I should get a flu shot in pregnancy | | | | | | | | | |  | |
| True | | 1 | 2 | 3 | 4 | 5 | 6 | 7 | False | Prefer not to answer | |
| 20. My family thinks that I should get a flu shot in pregnancy | | | | | | | | | |  | |
| True | | 1 | 2 | 3 | 4 | 5 | 6 | 7 | False | Prefer not to answer | |
| 21. My close friends think that I should get a flu shot in pregnancy | | | | | | | | | |  | |
| True | | 1 | 2 | 3 | 4 | 5 | 6 | 7 | False | Prefer not to answer | |
| 22. My faith leader thinks that I should get a flu shot in pregnancy | | | | | | | | | |  | |
| True | | 1 | 2 | 3 | 4 | 5 | 6 | 7 | False | Prefer not to answer | |
| 23. How much do you care what your doctor or midwife thinks you should do? | | | | | | | | | |  | |
| Very much | 1 | | 2 | 3 | 4 | 5 | 6 | 7 | Not at all | Prefer not to answer | |
| 24. How much do you care about what your partner thinks you should do? | | | | | | | | | |  | |
| Very much | 1 | | 2 | 3 | 4 | 5 | 6 | 7 | Not at all | Prefer not to answer | |
| 25. How much do you care what your family thinks you should do? | | | | | | | | | |  | |
| Very much | 1 | | 2 | 3 | 4 | 5 | 6 | 7 | Not at all | Prefer not to answer | |
| 26. How much do you care what your close friends think you should do? | | | | | | | | | |  | |
| Very much | 1 | | 2 | 3 | 4 | 5 | 6 | 7 | Not at all | Prefer not to answer | |
| 27. How much do you care what your faith leader thinks you should do? | | | | | | | | | |  | |
| Very much | 1 | | 2 | 3 | 4 | 5 | 6 | 7 | Not at all | Prefer not to answer | |
| 28. Getting the flu shot while pregnant is something most people I know | | | | | | | | | | |  |
| Definitely would do | | 1 | 2 | 3 | 4 | 5 | 6 | 7 | Definitely would not do | | Prefer not to answer |
| **How easy or hard is deciding to get a flu shot while pregnant?** | | | | | | | | | | |  |

| 29. Getting a flu shot while I am pregnant would be | | | | | | | | | | | | | | | | | |  | |
| --- | --- | --- | --- | --- | --- | --- | --- | --- | --- | --- | --- | --- | --- | --- | --- | --- | --- | --- | --- |
| Very easy | | 1 | | 2 | | 3 | | 4 | | 5 | | 6 | | 7 | | Very difficult | | Prefer not to answer | |
| 30. How likely is it that your doctor or midwife will talk with you about flu shots in pregnancy? | | | | | | | | | | | | | | | | | |  | |
| Very likely | | 1 | | 2 | | 3 | | 4 | | 5 | | 6 | | 7 | | Very unlikely | | Prefer not to answer | |
| 31. How likely is it that you will receive enough information about the flu shot in pregnancy? | | | | | | | | | | | | | | | | | |  | |
| Very likely | | 1 | | 2 | | 3 | | 4 | | 5 | | 6 | | 7 | | Very unlikely | | Prefer not to answer | |
| 32. How likely is it that you’ll make a definite decision about whether to get a flu shot this year? | | | | | | | | | | | | | | | | | |  | |
| Very likely | | 1 | | 2 | | 3 | | 4 | | 5 | | 6 | | 7 | | Very likely | | Prefer not to answer | |
| 33. How hard do you think it will be to get answers from your doctor or midwife about flu shots in pregnancy? | | | | | | | | | | | | | | | | |  | |  |
| Very hard | 1 | | 2 | | 3 | | 4 | | 5 | | 6 | | 7 | | Very easy | | Prefer not to answer | |  |
| 34. How hard do you think it will be to get enough information about flu shots in pregnancy? | | | | | | | | | | | | | | | | |  | |  |
| Very hard | 1 | | 2 | | 3 | | 4 | | 5 | | 6 | | 7 | | Very easy | | Prefer not to answer | |  |
| 35. How hard do you think it will be to make a decision about whether to get a flu shot in pregnancy? | | | | | | | | | | | | | | | | |  | |  |
| Very hard | 1 | | 2 | | 3 | | 4 | | 5 | | 6 | | 7 | | Very easy | | Prefer not to answer | |  |

**Please tell us a little about your information seeking and use.**

| 36. How often do people give you information about vaccines without you asking for it? | | | | | | | | | | | | | | | | | | | | | | | | | |  |
| --- | --- | --- | --- | --- | --- | --- | --- | --- | --- | --- | --- | --- | --- | --- | --- | --- | --- | --- | --- | --- | --- | --- | --- | --- | --- | --- |
| Very often | 1 | | | 2 | | | 3 | | | 4 | | | 5 | | | 6 | | | 7 | | | Never | | | | Prefer not to answer |
| 37. How often do you search for information about vaccines? | | | | | | | | | | | | | | | | | | | | | | | | | |  |
| Very often | 1 | | | 2 | | | 3 | | | 4 | | | 5 | | | 6 | | | 7 | | | Never | | | | Prefer not to answer |
| 38. How often do you ask other people for information about vaccines? | | | | | | | | | | | | | | | | | | | | | | | | | |  |
| Very often | 1 | | | 2 | | | 3 | | | 4 | | | 5 | | | 6 | | | 7 | | | Never | | | | Prefer not to answer |
| 39. How often do you learn things you weren’t looking for about vaccines from the news, television, or other traditional media? | | | | | | | | | | | | | | | | | | | | | | | | | |  |
| Very often | 1 | | | 2 | | | 3 | | | 4 | | | 5 | | | 6 | | | 7 | | | Never | | | | Prefer not to answer |
| 40. How often do you learn things you weren’t looking for about vaccines from the internet and social media? | | | | | | | | | | | | | | | | | | | | | | | | | |  |
| Very often | 1 | | | 2 | | | 3 | | | 4 | | | 5 | | | 6 | | | 7 | | | Never | | | | Prefer not to answer |
| 41. Have you ever used vaccination information to decide which vaccines to get for yourself? | | | | | | | | | | | | | | | | | | | | | | | | |  | |
| Very often | | 1 | | | 2 | | | 3 | | | 4 | | | 5 | | | 6 | | | 7 | | | Never | | Prefer not to answer | |
| 42. Have you ever used vaccination information to decide which vaccines to give a child? | | | | | | | | | | | | | | | | | | | | | | | | |  | |
| Very often | | 1 | | | 2 | | | 3 | | | 4 | | | 5 | | | 6 | | | 7 | | | Never | | Prefer not to answer | |
| 43. Have you ever used vaccination information to decide when to get shots for a child? | | | | | | | | | | | | | | | | | | | | | | | | |  | |
| Very often | | 1 | | | 2 | | | 3 | | | 4 | | | 5 | | | 6 | | | 7 | | | Never | | Prefer not to answer | |
| 44. Have you ever used vaccination information to decide where to go to get shots? | | | | | | | | | | | | | | | | | | | | | | | | |  | |
| Very often | | 1 | | | 2 | | | 3 | | | 4 | | | 5 | | | 6 | | | 7 | | | Never | | Prefer not to answer | |
| 45. In the past 12 months, how often have you looked for or asked for information about vaccines? | | | | | | | | | | | | | | | | | | | | | | | | |  | |
| Very often | | 1 | | | 2 | | | 3 | | | 4 | | | 5 | | | 6 | | | 7 | | | Never | | Prefer not to answer | |
| 46. How much would you say you know about vaccines overall? | | | | | | | | | | | | | | | | | | | | | | | | |  | |
| Everything | | | 1 | | | 2 | | | 3 | | | 4 | | | 5 | | | 6 | | | 7 | | | Nothing | Prefer not to answer | |
| 47. How much would you say you know about flu shots in particular? | | | | | | | | | | | | | | | | | | | | | | | | |  | |
| Everything | | | 1 | | | 2 | | | 3 | | | 4 | | | 5 | | | 6 | | | 7 | | | Nothing | Prefer not to answer | |
| 48. How much would you say you know about pregnancy in general? | | | | | | | | | | | | | | | | | | | | | | | | |  | |
| Everything | | | 1 | | | 2 | | | 3 | | | 4 | | | 5 | | | 6 | | | 7 | | | Nothing | Prefer not to answer | |
| 49. How much would you say you know about vaccines in pregnancy? | | | | | | | | | | | | | | | | | | | | | | | | |  | |
| Everything | | | 1 | | | 2 | | | 3 | | | 4 | | | 5 | | | 6 | | | 7 | | | Nothing | Prefer not to answer | |
| 50. How much would you say you know about influenza? | | | | | | | | | | | | | | | | | | | | | | | | |  | |
| Everything | | | 1 | | | 2 | | | 3 | | | 4 | | | 5 | | | 6 | | | 7 | | | Nothing | Prefer not to answer | |

51. From what sources, if any, do you currently get information on vaccination? Which do you find most trustworthy?

| Source | I consult this source | I think this source is most trustworthy |
| --- | --- | --- |
| Partner or spouse |  |  |
| Parents |  |  |
| Siblings |  |  |
| Other family |  |  |
| Friends |  |  |
| Family doctor |  |  |
| Obstetrician |  |  |
| Midwife |  |  |
| Doula |  |  |
| Walk-in Clinic |  |  |
| Public Health Clinic |  |  |
| Websites online |  |  |
| Social media sites or apps |  |  |
| Magazines |  |  |
| Television |  |  |
| Books |  |  |
| Pamphlets or brochures |  |  |
| Nurse |  |  |
| Pharmacist |  |  |
| Teacher/School |  |  |
| Movies |  |  |
| There is no good source |  |  |
| Other (please specify) |  |  |
| Prefer not to answer |  |  |

**Please tell us about yourself so we understand who is answering this survey.**

| 52. Did you get a flu shot last year? | | | | | |
| --- | --- | --- | --- | --- | --- |
| Yes | | Not Sure | | No | |
| 53. How often in the past 5 years have you gotten a flu shot? | | | | | |
| Every year | Most years | | Some years | A few years | Never |

54. How many weeks pregnant are you today? ___________

55. Which healthcare professional(s) follow(s) you during your pregnancy?

- Family physician
- Obstetrician / Gynecologist
- Midwife
- Nurse
- None of the above
- Prefer not to answer

56. How many previous births have you had?

- None: this will be my first
- 1
- 2
- 3
- 4 or more previous children
- Prefer not to answer

57. How many children under the age of 19 years live in your household? ____________ Prefer not to answer

58. What is your age?

- 18-24 years
- 25-29
- 30-34
- 35-39
- 40-44
- 45 or older
- Prefer not to answer

59. In which province or territory do you live?

- British Columbia (BC)
- Alberta (AB)
- Saskatchewan (SK)
- Manitoba (MB)
- Ontario (ON)
- Quebec (QC)
- New Brunswick (NB)
- Nova Scotia (NS)
- Prince Edward Island (PE)
- Newfoundland (NF)
- Northwest Territories (NT)
- Yukon (YK)
- Nunavut (NU)
- Prefer not to answer

60. What are the first 3 digits of your postal code? ___ ___ ___ Prefer not to answer

61. How would you describe your ancestry? If you have more than one ancestry, please check any that apply.

|  | Western European |
| --- | --- |
|  | Eastern European |
|  | Caribbean |
|  | African |
|  | Chinese |
|  | South Asian (*i.e. Indian, Pakistani, Sri Lankan*) |
|  | Filipino |
|  | SouthEast Asian (*i.e. Vietnemese, Cambodian*) |
|  | Arab |
|  | West Asian (*i.e Iranian, Afghan*) |
|  | Latin American |
|  | Korean |
|  | Japanese |
|  | First Nations |
|  | Inuit |
|  | Metis |
|  | Other (please specify) |
|  | Prefer not to answer |

62. Do you have any chronic health conditions (e.g., asthma, diabetes, heart condition)

- Yes
- No

63. Please select the option below that best describes your highest level of education achieved:

|  | Elementary/middle school |
| --- | --- |
|  | Some secondary school |
|  | Completed secondary |
|  | Some college/university |
|  | Completed college/university |
|  | Some post-graduate |
|  | Completed post-graduate |
|  | Prefer not to answer |

64. Please select the option below that best describes your living situation over the last three months.

|  | I live at home with my parents/guardians |
| --- | --- |
|  | I live with a roommate(s) |
|  | I live with a husband/partner/common-law |
|  | I live alone with child/children |
|  | I live alone |
|  | Other (please specify) ______________________________ |
|  | Prefer not to answer |

65. Where were you born?

- Canada
- Outside Canada: Specify country__________
- Prefer not to answer

66. Please select the option below that best describes your employment status over the last three months.

|  | I work part time |
| --- | --- |
|  | I work full time |
|  | I’m not working |
|  | Prefer not to answer |

67. Which of the following best describes your annual household income…?

|  | Less than $20,000 |
| --- | --- |
|  | $20,000 to $39,999 |
|  | $40,000 to $59,999 |
|  | $60,000 to $79,999 |
|  | $80,000 to $99,999 |
|  | $100,000+ |
|  | Prefer not to answer |

**Thank you for taking the time to answer this survey!**

**Survey 2**

| 1 | Did you receive a flu shot this past fall/winter (2017-2018)? | | | |
| --- | --- | --- | --- | --- |
|  | YES | NO | Unsure | Prefer not to answer |
| 1a | If YES to Q1: Were you pregnant when you received the shot? | | | |
|  | YES | NO | Unsure | Prefer not to answer |
| 1b | If YES to Q1: Why DID you get the flu shot? (check all that apply) | | | |
|  | - I got the flu shot while pregnant because a health professional recommended it | | | |
|  | - It was convenient to get the flu shot | | | |
|  | - I felt the flu shot was worthwhile to protect my own health | | | |
|  | - I felt the flu shot was worthwhile to protect my baby’s health | | | |
|  | - I felt the flu shot works | | | |
|  | - I felt the flu shot was safe | | | |
|  | - I felt well-informed about the flu shot | | | |
|  | - My partner wanted me to get the flu shot while pregnant | | | |
|  | - My family (other than partner) wanted me to get the flu shot | | | |
|  | - My friends wanted me to get the flu shot | | | |
|  | - I wanted to protect the health of my family | | | |
|  | - I wanted to protect the health of others in general | | | |
|  | - I felt pressure to get the flu shot (please specify from whom you felt pressure):________ _______________________________ | | | |
|  | - There are other reasons I got the flu shot (please tell us what reasons): ____________ __________________________________________________________ | | | |
|  | - Prefer not to answer | | | |

**Please think back over the past 6 months and let us know what you decided about getting shots in pregnancy.**

| 1c | If NO to Q1: Why did you NOT get the flu shot? (check all that apply) |
| --- | --- |
|  | - I had difficulty getting an appointment |
|  | - I didn’t think I would get sick with the flu |
|  | - I thought my body would fight the flu without the vaccine |
|  | - I feel the flu vaccine does not work |
|  | - I felt the flu shot wasn’t needed to protect my health |
|  | - I thought the flu shot would make me sick with the flu |
|  | - I was worried about possible side effects (or harm from) the flu shot on me |
|  | - I was worried about possible side effects (or harm from) the flu shot on my baby |
|  | - I did not have enough information to decide to be vaccinated against the flu |
|  | - A health professional advised me not to get the flu shot while pregnant (please specify what kind of health professional): ___________________________________________ |
|  | - My partner did not want me to get the flu shot while pregnant |
|  | - My family (other than partner) did not want me to get the flu shot |
|  | - My friends did not want me to get the flu shot |
|  | - I don’t like needles |
|  | - As a general rule I do not get shots (vaccines) |
|  | - There are other reasons I did not get the flu shot (please tell us what reasons):____________ ______________________________________________________________ |

|  | - Prefer not to answer | | | | | | | | | | | | | | | | |
| --- | --- | --- | --- | --- | --- | --- | --- | --- | --- | --- | --- | --- | --- | --- | --- | --- | --- |
| 2 | Did you receive any other shots (vaccines) during your current/recent pregnancy? | | | | | | | | | | | | | | | | |
|  | YES | | | NO | | | | | | Unsure | | | | Prefer not to answer | | | |
|  |  | | |  | | | | | |  | | | |  | | | |
| 2a | If YES to Q2: Which vaccine(s) did you receive in pregnancy? | | | | | | | | | | | | | | | | |
|  | Influenza  (Flu shot) | TDaP  (Tetanus, Diphtheria, Pertussis aka Whooping Cough) | | | | HepB  (Hepatitis B) | | HPV  (Human papilloma virus) | | | | Other:_____________(Please specify which) | | | | Don’t’ know | |
|  | **Now, please tell us a little bit about your thoughts and plans about shots for your new baby:** | | | | | | | | | | | | | | | | |
| 3 | What are your plans for vaccinations and your new baby? | | | | | | | | | | | | | | | | |
|  | I plan to vaccinate fully on schedule | | | | I plan to vaccinate with a selective or delayed schedule | | | | | | I am not planning to vaccinate my baby at this time | | | | Prefer not to answer | | |
| 4 | Has your new baby received their 2 month shots? | | | | | | | | | | | | | | | | |
|  | Yes | | Some:  My baby got some of the 2 month shots, but not all | | | | No:  My baby is not yet 2 months old | | No:  I am using an alternative schedule for infant shots | | | | No: My baby will not be getting these shots | | | | Prefer not to answer |

| 5. Getting my baby all the recommended shots on time would be | | | | | | | | |  |
| --- | --- | --- | --- | --- | --- | --- | --- | --- | --- |
| Very good | 1 | 2 | 3 | 4 | 5 | 6 | 7 | Very Bad | Prefer not to answer |
| 6. People I care about think I should get my baby all the recommended shots on time | | | | | | | | |  |
| True | 1 | 2 | 3 | 4 | 5 | 6 | 7 | False | Prefer not to answer |
| 7. Getting all the recommended shots in time for my baby would be | | | | | | | | |  |
| Very easy | 1 | 2 | 3 | 4 | 5 | 6 | 7 | Very hard | Prefer not to answer |
