# Supplementary material for: Understanding Influenza Vaccination During Pregnancy in Canada: Attitudes, Norms, Intentions, and Vaccine Uptake
Source: Health Educ Behav. 2021 Apr 17;48(5):680–9. doi: 10.1177/10901981211001863 (PMC12675836; doi:10.1177/10901981211001863)
Supplement: sj-docx-2-heb-10.1177_10901981211001863 – Supplemental material for Understanding Influenza Vaccination During Pregnancy in Canada: Attitudes, Norms, Intentions, and Vaccine Uptake [file sj-docx-2-heb-10.1177_10901981211001863.docx]

**Appendix B. Model Constructs and Univariate Correlation Analysis Results**

| Model  Construct | | Construct Variable | | Question | Variable mean scores (scale 1-7) | Construct standardized Cronbach’s alpha | R-square | Univariate correlation estimate | Univariate 95% confidence intervals | Univariate p-value |
| --- | --- | --- | --- | --- | --- | --- | --- | --- | --- | --- |
| Outcome | | Intention | | 1: I will get a flu shot this season while I am pregnant. [very likely-very unlikely] | 4.25 | n/a | n/a | n/a | n/a | n/a |
| Beliefs | | Combined Attitudes | | 2: Getting a flu shot while I am pregnant would be [very good-very bad]  3: Getting a flu shot while I am pregnant would be [wise-foolish]  4: Getting a flu shot while I am pregnant is [necessary-unnecessary] | 3.90  3.75  4.03 | 0.95 | 0.7281 | 1.02 | 0.97 – 1.07 | < 0.001 |
|  |  | **Combined Behavioral Beliefs*** | | **5: Getting a flu shot while I am pregnant would help me avoid the flu**  **7: Getting a flu shot while I am pregnant would help protect other people in my community**  **8: Getting a flu shot while I am pregnant would make me sick**  **9: Getting a flu shot while I am pregnant could be bad for my baby**  **10: How likely is it that I will get the flu while pregnant?**  **[very likely-very unlikely]**  **11: Avoiding the flu while pregnant would be**  **13: Protecting other people in my community from the flu would be**  **14: Having a side effect from the flu shot would be**  **15: My baby being harmed by something I did in pregnancy would be**  **16: Getting the flu while pregnant would be**  **[very good-very bad]** | **3.79**  **3.63**  **4.05**  **3.90**  **4.11**  **2.36**  **2.33**  **5.74**  **6.23**  **5.34** | **0.65** | **0.4700** | **0.21** | **0.19 – 0.23** | **< 0.001** |
| **Norms** | | **Direct Norms** | | **17: Most people who are important to me think I should get vaccinated against the flu during my pregnancy [true-false]** | **4.11** | **n/a** | **0.3953** | **0.69** | **0.62 – 0.76** | **< 0.001** |
|  |  | **Descriptive Norms** | | **28: Getting the flu shot while pregnant is something most people I know [definitely would do-definitely would not do]** | **3.85** | **n/a** | **0.3223** | **0.76** | **0.67 – 0.84** | **< 0.001** |
| **Control** | | **Control** | | **29: Getting a flu shot while I am pregnant would be [very easy-very hard]** | **3.25** | **n/a** | **0.1932** | **0.52** | **0.44 – 0.61** | **< 0.001** |
| **Information**  **Behavior** | | **Information Seeking** | | **37: How often do you search for information about vaccines?**  **38: How often do you ask other people for information about vaccines?**  **45: In the past 12 months, how often have you looked for or asked for information about vaccines?**  **[very often-never]** | **4.16**  **4.53**  **4.15** | **0.83** | **0.0001** | **0.47** | **0.35 – 0.58** | **< 0.001** |
|  |  | **Information Use** | | **41: Have you ever used vaccination information to decide which vaccines to get for yourself?**  **42: Have you ever used vaccination information to decide which vaccines to give a child?**  **43: Have you ever used vaccination information to decide when to get shots for a child?**  **[very often-never]** | **3.81**  **3.55**  **3.48** | **0.86** | **0.0093** | **0.25** | **0.14 – 0.37** | **< 0.001** |
|  |  | **Knowledge** | | **46: How much would you say you know about vaccines overall?**  **47: How much would you say you know about flu shots in particular?**  **49: How much would you say you know about vaccines in pregnancy?**  **50: How much would you say you know about influenza?**  **[everything-nothing]** | **3.80**  **3.82**  **4.07**  **3.88** | **0.86** | **0.0022** | **0.44** | **0.30 – 0.58** | **< 0.001** |
| **Demographics** | | **Last year Behavior (Yes vs No)** | | **52: Did you get a flu shot last year?** | **n/a** | **n/a** | **0.0005** | **-2.65** | **-2.96 – -2.34** | **< 0.001** |
|  |  | **Last year Behavior (Not sure vs No)** | |  |  |  |  | **-1.24** | **-1.97 – -0.50** | **0.001** |
|  |  | **Past 5 years Behavior (Every year vs Never)** | | **53: How often in the past 5 years have you gotten a flu shot?** | **n/a** | **n/a** | **0.0027** | **-3.32** | **-3.75 – -2.88** | **< 0.001** |
|  |  | **Past 5 years Behavior (Most-some-few years vs Never)** | |  |  |  |  | **-2.22** | **-2.57 – -1.88** | **< 0.001** |
|  |  | Weeks pregnant | | 54: How many weeks pregnant are you today? | 17.33 | n/a | 0.0007 | -0.01 | -0.02 – 0.01 | 0.505 |
|  |  | Previous births (1 vs 0) | | 56: How many previous births have you had? | n/a | n/a | 0.0030 | -0.04 | -0.45 – 0.38 | 0.865 |
|  |  | Previous births (2 vs 0) | |  |  |  |  | 0.09 | -0.50 – 0.68 | 0.763 |
|  |  | Previous births (3 vs 0) | |  |  |  |  | 0.71 | -0.15 – 1.56 | 0.104 |
|  |  | Previous births (4+ vs 0) | |  |  |  |  | 0.39 | -0.67 – 1.44 | 0.470 |
|  |  | Age (25-29 vs 18-24yr) | | 58: What is your age? | n/a | n/a | 0.0041 | 0.01 | -0.51 – 0.52 | 0.977 |
|  |  | Age (30-34 vs 18-24yr) | |  |  |  |  | -0.29 | -0.82 – 0.23 | 0.280 |
|  |  | Age (35-39 vs 18-24yr) | |  |  |  |  | -0.21 | -0.77 – 0.35 | 0.465 |
|  |  | Age (40-44 vs 18-24yr) | |  |  |  |  | -0.69 | -1.63 – 0.25 | 0.148 |
|  |  | **Education (College/university vs Secondary or less)** | | **63: Please select the option below that best describes your highest level of education achieved** | **n/a** | **n/a** | 0.011320 | **-0.66** | **-1.16 – -0.16** | **0.010** |
|  |  | Education (Post-graduate vs Secondary or less) | |  |  |  |  | -0.45 | -1.02 – 0.11 | 0.118 |
|  |  | Where born (Canada vs other) | | 65: Where were you born? | n/a | n/a | 0.0003 | 0.10 | -0.40 – 0.61 | 0.684 |
|  |  | Working (part time vs not working) | | **66: Please select the option below that best describes your employment status over the last three months** | n/a | n/a | 0.011767 | -0.41 | -0.96 – 0.14 | 0.143 |
|  |  | **Working (full time vs not working)** | |  |  |  |  | **-0.62** | **-1.08 – -0.16** | **0.009** |
|  | | Income | | 67 ($60,000+ vs < $60,000)^***^ | n/a | n/a | 0.0050 | 0.59 | 0.24 – 1.46 | 0.256 |
|  |  | | ** bolding indicates statistical significance in univariate analysis*  ***no mean is reported for questions answered categorically rather than on a 1-7 rating scale*  **** Prefer not to answer (n = 3) combined with $60,000+* | | | | | | | |
